# Structural properties of [2Fe-2S] ISCA2-IBA57: a complex of the mitochondrial iron-sulfur cluster assembly machinery

**DOI:** 10.1038/s41598-019-55313-5

**Published:** 2019-12-12

**Authors:** Veronica Nasta, Stefano Da Vela, Spyridon Gourdoupis, Simone Ciofi-Baffoni, Dmitri I. Svergun, Lucia Banci

**Affiliations:** 1 Magnetic Resonance Center CERM, University of Florence, Via Luigi Sacconi 6, 50019 Sesto Fiorentino, Florence Italy; 2 Department of Chemistry, University of Florence, Via della Lastruccia 3, 50019 Sesto Fiorentino, Florence Italy; 3 European Molecular Biology Laboratory, Hamburg Outstation, EMBL c/o DESY, Notkestrasse 85, 22607 Hamburg, Germany

**Keywords:** SAXS, Bioinformatics, Bioinorganic chemistry, Metals

## Abstract

In mitochondria, a complex protein machinery is devoted to the maturation of iron-sulfur cluster proteins. Structural information on the last steps of the machinery, which involve ISCA1, ISCA2 and IBA57 proteins, needs to be acquired in order to define how these proteins cooperate each other. We report here the use of an integrative approach, utilizing information from small-angle X-ray scattering (SAXS) and bioinformatics-driven docking prediction, to determine a low-resolution structural model of the human mitochondrial [2Fe-2S]^2+^ ISCA2-IBA57 complex. In the applied experimental conditions, all the data converge to a structural organization of dimer of dimers for the [2Fe-2S]^2+^ ISCA2-IBA57 complex with ISCA2 providing the homodimerization core interface. The [2Fe-2S] cluster is out of the ISCA2 core while being shared with IBA57 in the dimer. The specific interaction pattern identified from the dimeric [2Fe-2S]^2+^ ISCA2-IBA57 structural model allowed us to define the molecular grounds of the pathogenic Arg146Trp mutation of IBA57. This finding suggests that the dimeric [2Fe-2S] ISCA2-IBA57 hetero-complex is a physiologically relevant species playing a role in mitochondrial [4Fe-4S] protein biogenesis.

## Introduction

Iron-sulfur (Fe-S) clusters are among the most ancient inorganic protein co-factors^1^. The most common Fe-S clusters found in nature are [2Fe-2S] and [4Fe-4S] clusters, which are formed by tetrahedrally coordinated iron atoms with bridging sulfides and are coordinated to the protein in the majority of cases through cysteine residues^2^. These protein-bound clusters are essential players in a variety of biological processes ranging from electron transfer to enzymatic reactions^3,4^. Despite their simple chemical composition, the biosynthesis of Fe-S clusters is a complex and strictly regulated process involving multiple protein components^4–6^. In mitochondria, a complex protein machinery is devoted to the maturation of iron-sulfur cluster (ISC)-containing proteins and enzymes^7,8^. In a first step, this machinery, named mitochondrial ISC assembly machinery hereafter, assembles a [2Fe-2S] cluster^9^. In a second step, the machinery combines two [2Fe-2S] clusters to assemble a [4Fe-4S] cluster, which is then inserted into mitochondrial target proteins^10,11^. The molecular events at the basis of this second step are still quite elusive. Some of the members of this second step include the A-type ISC proteins, monothiol glutaredoxins and the IBA57 protein family. The human genome encodes, among the proteins localized in mitochondria, two A-type ISC proteins, termed ISCA1 and ISCA2, a monothiol glutaredoxin, GLRX5, and a IBA57 protein. GLRX5 works as a [2Fe-2S] cluster transfer protein in the mitochondrial matrix^12,13^. ISCAs and IBA57 proteins are required for the maturation of mitochondrial [4Fe-4S] proteins^11,14,15^, but their interaction network is still argument of debate in the literature. In *S. cerevisiae* it has been proposed that these proteins act in the same biochemical pathway, which encompasses a hetero complex formed by *S. cerevisiae* ISCA1, ISCA2, and IBA57 homologues as the functional unit devoted to convert two [2Fe-2S] clusters^11,16^, received by *S. cerevisiae* GLRX5 homologue^13,17^, to a [4Fe-4S] cluster^18,19^. On the contrary, data collected on human cells suggested that a ternary complex among ISCA1, ISCA2, and IBA57 is not operative in the physiological state of human cells^20^. This model was based on the findings that, although ISCA1 and ISCA2 reciprocally interact, only ISCA2, and not ISCA1, was found to interact with IBA57, at variance with *S. cerevisiae* data^20^. Recently, we found that the three proteins GLRX5, ISCA2 and IBA57 lead to the formation of a [2Fe-2S]^2+^ ISCA2-IBA57 complex *in vitro* ([2Fe-2S] ISCA2-IBA57 hereafter)^21^. Specifically, the latter complex is formed in two steps: first, the [2Fe-2S] cluster is transferred from [2Fe-2S] GLRX5 to apo ISCA2 and then IBA57 interacts with [2Fe-2S] ISCA2 forming the heterodimeric [2Fe-2S] ISCA2-IBA57 complex. The latter complex was not formed with ISCA1^21^, supporting the model that, in humans, ISCA proteins modulate their interactions network in a complex and dynamic manner. The [2Fe-2S] ISCA2-IBA57 complex is asymmetrically coordinated by the conserved cysteines Cys 79, Cys 144, and Cys 146 of ISCA2 and by Cys 259 from IBA57, thus resulting the [2Fe-2S] cluster bridged between the two proteins^21^. We also showed that cluster binding is required to promote complex formation between ISCA2 and IBA57 proteins^21^, as indeed the apo proteins do not interact. Finally, each of the four cysteines are essential to induce the [2Fe-2S] hetero-complex formation, since, once individually mutated to Ala, the [2Fe-2S] ISCA2-IBA57 complex formation is abolished^21^. Overall, these data indicate that ISCA2, and not ISCA1, specifically interacts with IBA57 to form a cluster-mediated interaction.

Mutations in ISCA2 and IBA57 genes have been reported in patients affected by multiple mitochondrial dysfunctions syndromes (MMDS), specifically MMDS3 for IBA57^22–28^ and MMDS4 for ISCA2^29,30^. In these patients, the biochemical phenotype is similar and was characterized by a defect in the respiratory chain complexes I and II and a decrease in mitochondrial protein lipoylation, both effects resulting from impaired assembly of [4Fe-4S] clusters^25,26,31^. It was also reported that the expression of ISCA2 is drastically decreased in IBA57 patients as well as ISCA2 patients have a decreased amount of IBA57^32^, at support of a direct functional link of these two proteins^26^. All these data indicate, in agreement with the *in vitro* [2Fe-2S] ISCA2-IBA57 complex formation^21^, that ISCA2 and IBA57 operate in a synergistic way in the maturation of [4Fe-4S] proteins.

We report here the use of an integrative approach, utilizing information from small-angle X-ray scattering (SAXS) and bioinformatics-driven docking prediction to determine a low-resolution structural model of the human mitochondrial [2Fe-2S] ISCA2-IBA57 complex. Specifically, the SAXS data were combined with mutagenesis data and bioinformatic prediction-driven modelling to derive the model of the [2Fe-2S] ISCA2-IBA57 complex. Based on this structural model, we identified the specific [2Fe-2S] ISCA2-IBA57 interaction pattern, which allow us to rationalize the role of the pathogenic mutation Arg146Trp in IBA57.

## Results

### Structural information on IBA57 and apo ISCA2 by SAXS

**Figure 1:**
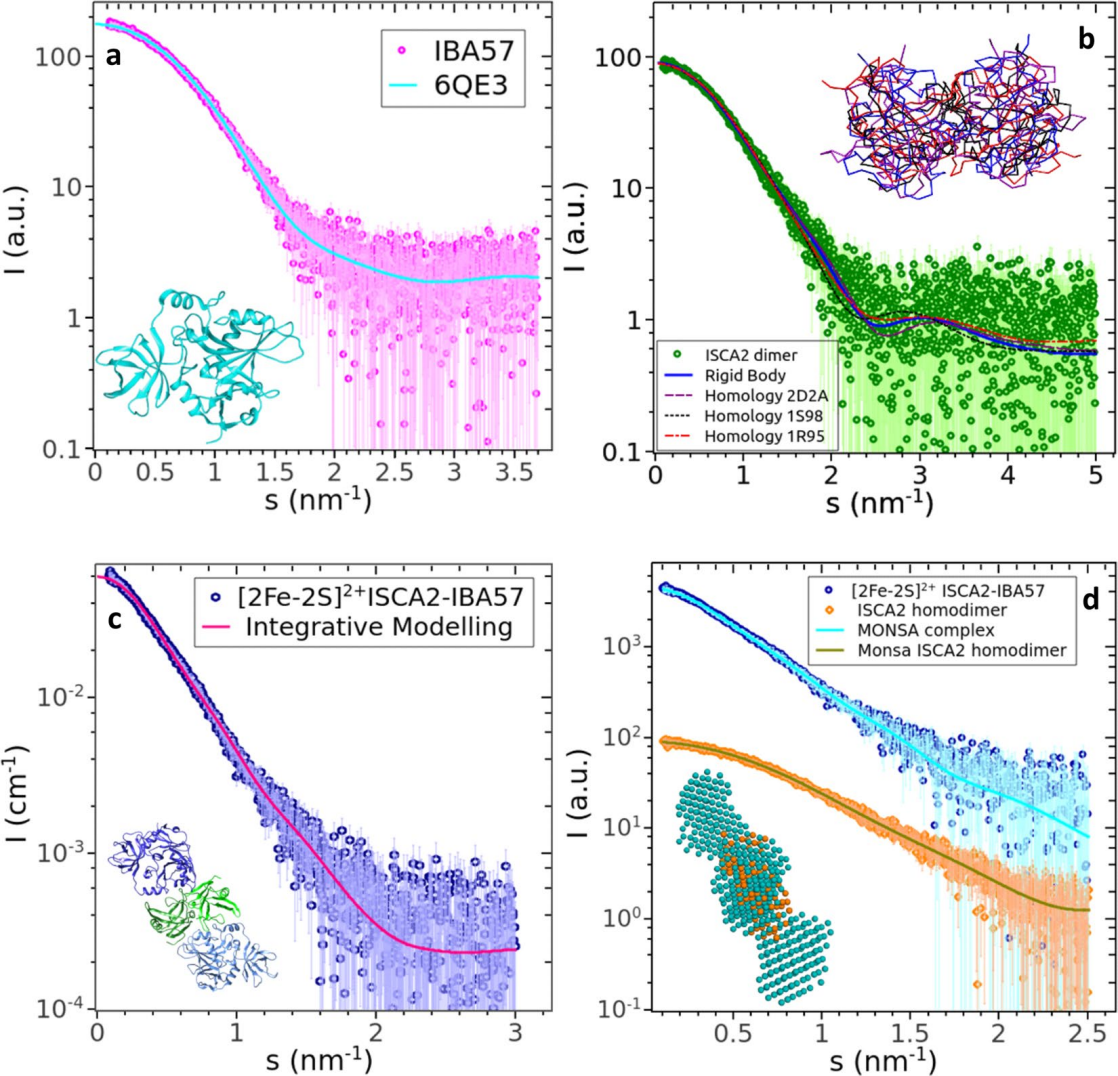
SAXS curves and curve fitting of IBA57, apo ISCA2 and [2Fe-2S]^2+^ ISCA2-IBA57. (**a**) SAXS profile of IBA57 and the fit computed from the crystal structure 6QE3 (ribbon diagram in the inset). (**b**) SAXS profile of ISCA2 homodimer and fits by rigid body and homology models. Inset: backbones of the fitted dimers showing similar overall architecture, superimposed with SUPCOMB^67^, with the same color-coding as the fit curves. (**c**) Rigid body fit using cluster 1 heterodimers. Inset: the model of the complex in which dimerization is mediated by ISCA2. (**d**) Simultaneous multiphase *ab initio* fit to the complex and to the ISCA2 homodimer. Inset: two-phase model; orange beads, ISCA2 homodimer, teal beads, the complex. The scattering intensity is displayed as a function of the momentum transfer *s* = 4π sin(θ)/λ, where 2θ is the scattering angle and λ is the X-ray wavelength.

The SAXS data on IBA57 suggested that the crystal structure (6QE3.pdb)^21,33^ is maintained in solution and the scattering computed from this structure fits well the experimental SAXS profile of IBA57 (discrepancy χ^2^ = 1.075, continuous line in Fig. 1a).

**Figure 2:**
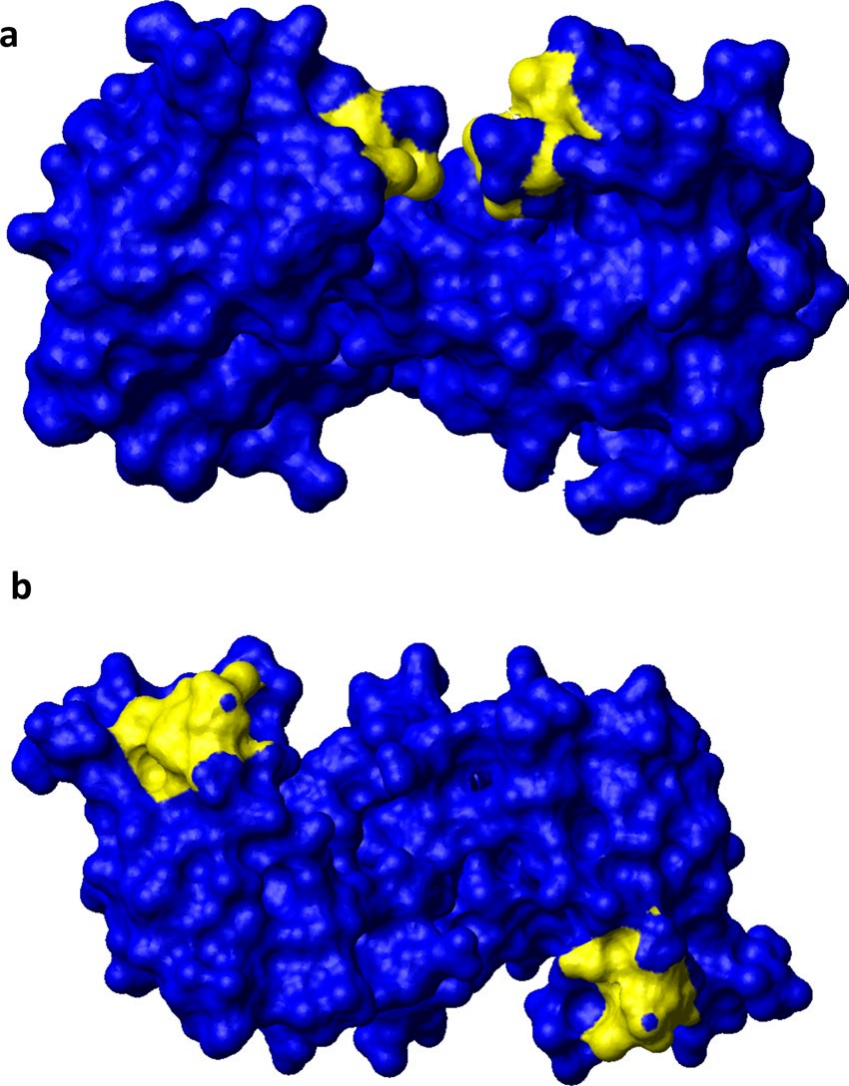
Structural models of apo ISCA2. Contact surface of dimeric apo ISCA2 obtained by the crystal structure of homodimeric SufA (PDB ID: 2D2A) (**a**) and by rigid body modelling assuming P2 symmetry (**b**). The yellow regions identify the Cys ligands.

For the apo ISCA2 sample, the SAXS curve from the main chromatographic SEC-SAXS peak is not consistent with a monomeric 12.6 kDa protein. Instead, the overall parameters presented in Table 1 point to the presence of an apo ISCA2 homodimer in solution, consistently with previous NMR data that showed a dimeric state for ISCA2 in both apo and holo forms^18^. To model the dimer, two different approaches were followed. In the first approach, the experimentally validated structure of the ISCA2 monomer obtained with Modeller (see *Structural Coordinates* paragraph of the *Methods* section) was used for rigid body modelling of the ISCA2 dimer assuming a P2 symmetry (see *Methods*). The best dimeric model (blue continuous line in Fig. 1b) gave a final χ2 of 1.063 against the SAXS curve of apo ISCA2 (green dots). In the second approach, ISCA2 dimers were generated by superimposing the Modeller monomer on existing crystal structures of homodimers of bacterial ISCA homologues (PDB: 2D2A, 1S98, 1R95). These dimeric structures were used to fit the SAXS curve of apo ISCA2 resulting in χ2 values of 1.049 for 2D2A, 1.216 for 1S98 and 1.136 for 1R95 (Fig. 1b). All the dimer models obtained with the two approaches fitted well to the experimental SAXS patterns and revealed an overall low-resolution “8”-like shape (Fig. 1b, inset), but with different dimeric interfaces. Indeed, the two best models, with comparable χ2 values of 1.049 and 1.063, were obtained with structures having the dimeric interfaces differently exposing the cysteine ligands on the two subunits of the dimer. In the structure based on SufA bacterial homologue (χ2 value of 1.049), the three cysteine ligands face each other from the two subunits of the dimer (Fig. 2a). On the contrary, in the rigid body modelling structure providing the χ2 value of 1.063, the cysteine ligands of the two subunits are solvent exposed on the opposite side of the dimer (Fig. 2b). These two models yield very similar fits to the SAXS data (see Discussion).

**Table 1:** Overall parameters from the SAXS profiles used for the modeling.

Data collection parameters	Robotic sample changer	SEC-SAXS	
Radiation source	PETRA III (DESY)	PETRA III (DESY)	
Beamline	EMBL P12	EMBL P12	
Detector	Pilatus 6 M	Pilatus 6 M	
Wavelength	0.124 nm	0.124 nm	
Sample-to-detector distance	3.1 m	3.1 m	
*s* range	0.017–7.3 nm^−1^	0.026–7.3 nm^−1^	
Exposure time	40 × 0.1 s	1 s/frame	
Temperature	293.2 K	293.2 K	
**Parameter (unit)**	**IBA57**	**(ISCA2)**_**2**_	**(ISCA2-IBA57)**_**2**_
Rg^a^ Guinier (nm)	2.16 ± 0.02	2.03 ± 0.05	3.95 ± 0.12
Rg PDDF^b^ (nm)	2.17	2.07	4.03
Dmax^c^ (nm)	7.1 ± 0.1	6.7 ± 0.1	12.4
Vp^d^ (nm^3^)	66 ± 10	25 ± 10	168 ± 10
Mr I(0)	n/a	n/a	79900
Mr^e^ (from Vp)	41300 ± 8260	15400 ± 3080	105000 ± 21000
Mr Bayes^66^	25250–29250	17750–20250	61600–75300
Mr sequence	34925	25130	101660

^a^Rg: radius of gyration.

^b^PDDF pair distance distribution function.

^c^Dmax: maximal intramolecular distance.

^d^Vp: particle volume. ^e^Mr relative molar mass.

### Structural model of [2Fe-2S] ISCA2-IBA57 complex

A starting structural model of the [2Fe-2S] ISCA2-IBA57 complex was first calculated by combining bioinformatics interface predictions with information-driven docking. This approach has already been applied with success to model protein-protein complexes whose interaction is metal-mediated^34^, a circumstance that applies to the formation of the [2Fe-2S] ISCA2-IBA57 complex^21^.

**Figure 3:**
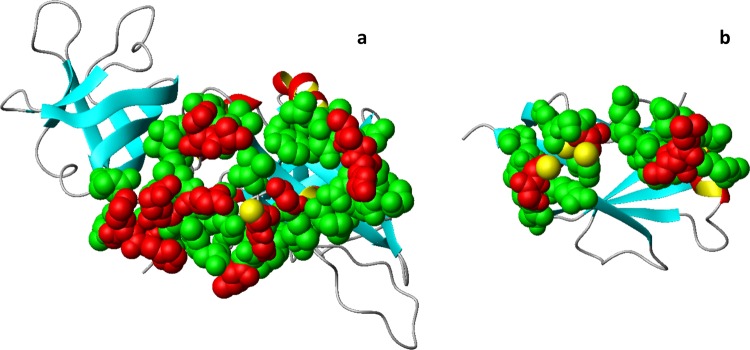
Predicted interacting residues of IBA57 and ISCA2 mapped on the respective structures, obtained by the interface prediction program WHISCY and by the protein-protein docking program HADDOCK. Space filling representation of active (red) and passive (green) residues used for docking (see Supplementary Table S1 for the corresponding residue numbers) on the ribbon diagram of IBA57 (**a**) and ISCA2 (**b**). Active residues are defined based on interface predictions using WHISCY, passive residues are surface neighbors of active residues defined by HADDOCK (see Methods). Yellow spheres indicate the Sγ of Cys ligands, i.e. Cys 259 in IBA57 and Cys 79, Cys 144 and Cys 146 in ISCA2.

**Figure 4:**
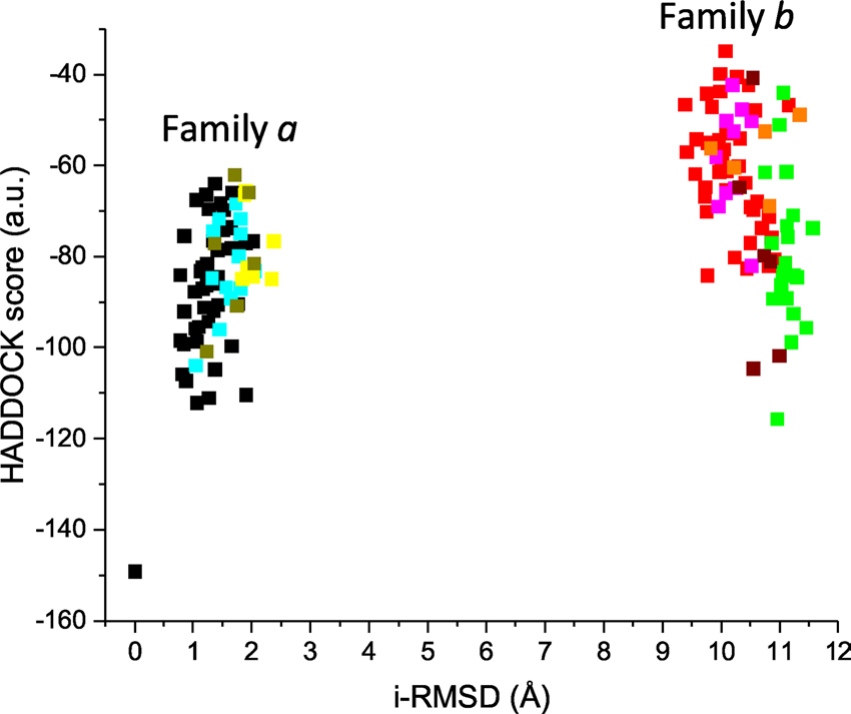
Clustering the models calculated by HADDOCK on the basis of common contacts. The plot is based on water-refined models generated by HADDOCK runs. The clusters (indicated with different colors in the plot) are based on the interface-ligand RMSDs calculated by HADDOCK, with the interface defined automatically based on all observed contacts. The clusters result and grouped in two families, *a* and *b*. i-RMSD indicates the interface-RMSD calculated on the backbone (CA, C, N, O) atoms of all residues involved in intermolecular contact using a 10 Å cutoff. This structural analysis was made with respect to the best HADDOCK model (the one with the lowest HADDOCK score).

**Figure 5:**
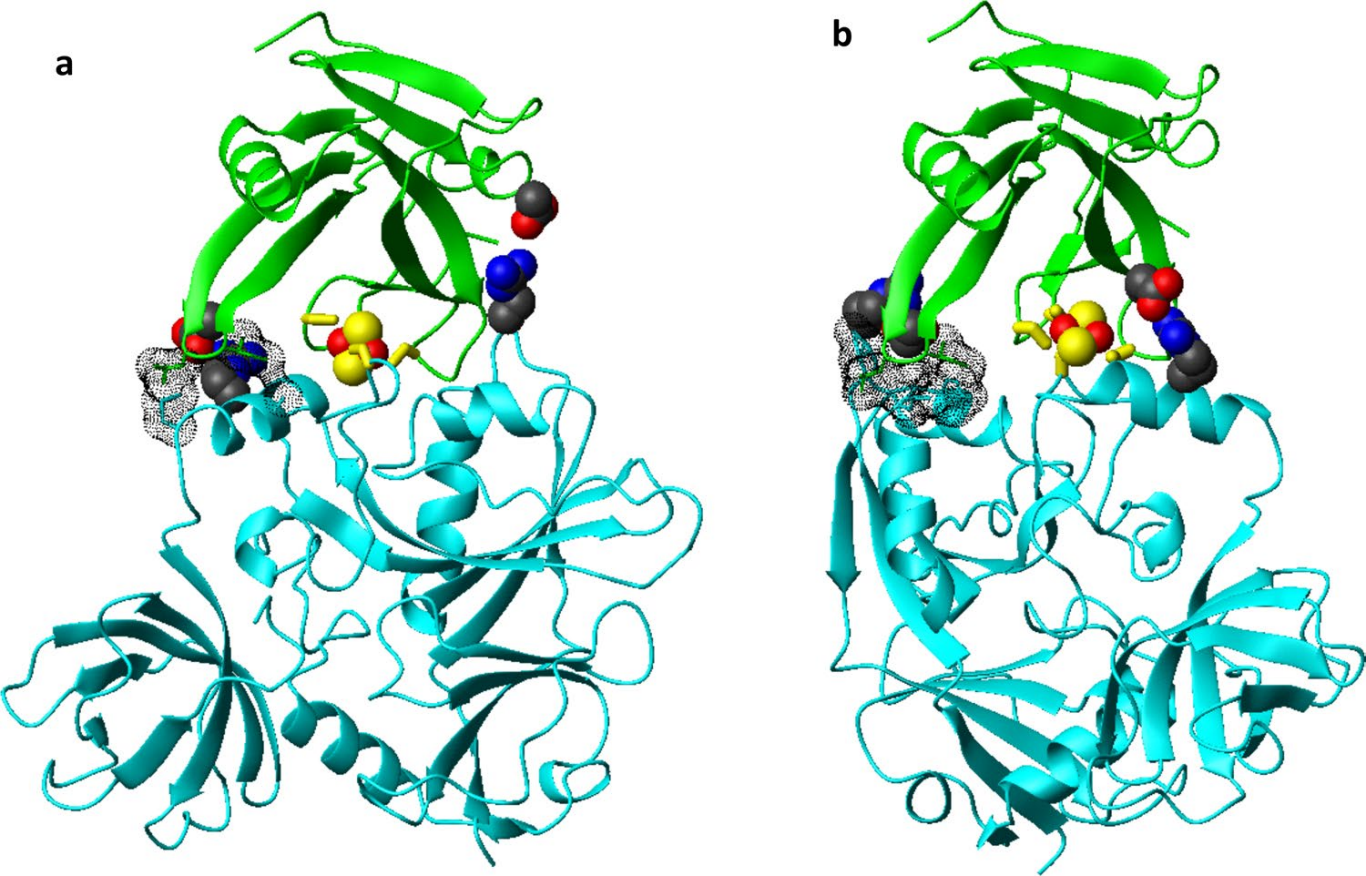
Structural models of the heterodimeric [2Fe-2S] ISCA2-IBA57 complex obtained by HADDOCK. Ribbon diagram of the best scoring HADDOCK model of [2Fe-2S] ISCA2-IBA57 in family *a* (from cluster 1) (**a**) and in family *b* (from cluster 3) (**b**). IBA57 is in cyan, ISCA2 is in green. The Cys ligands are shown as yellow stick, the iron and sulfur atoms of the [2Fe-2S] cluster are in red and yellow spheres, respectively. Intermolecular electrostatic interactions between Asp 111 (ISCA2) and Arg 146 (IBA57) and between Glu 126 (ISCA2) and Arg 268 (IBA57) in (**a**), and between Glu 75 (ISCA2) and Arg 268 (IBA57) and between Glu 126 (ISCA2) and Arg 146 (IBA57) in (**b**) are shown in CPK mode. Hydrophobic contacts between Ile 128 (ISCA2) and Ala 267 (IBA57) and between Leu 127 (ISCA2) and Met 272 (IBA57) in (**a**), and between Leu 127 (ISCA2), Ile 128 (ISCA2), Leu 142 (IBA57), Tyr 143 (IBA57), Ile 145 (IBA57) in (**b**) are shown in dots. The side-chains of the latter residues are shown as sticks.

While the structure of IBA57 is available^21,33^, the structure of human [2Fe-2S]-bound ISCA2 is not. However, a [2Fe-2S] cluster-bound crystal structure of IscA from *Thermosynechococcus elongatus* has been solved^35^. In this structure, one partially exposed [2Fe-2S] cluster is coordinated by two conformationally distinct IscA molecules. An asymmetric cysteinyl ligation occurs via the conserved cysteines Cys 37, Cys 101, Cys 103 from one molecule and Cys103 from the other, with the latter molecule providing only one cysteine ligand to cluster coordination. This cysteine has been shown to have more conformational flexibility than the other coordinating cysteines provided by the other protein molecule^35^. Considering that we have previously showed that ISCA2 coordinates the cluster in the [2Fe-2S] ISCA2-IBA57 complex with the same three Cys as found in the first molecule of the bacterial dimer^21^, we modelled human ISCA2 sequence on this molecule of IscA from *T. elongates*. The obtained structural model of human ISCA2 has a cluster coordination where an iron ion is coordinated by Cys 144 and Cys 146 and the other iron ion is coordinated by Cys 79 ([2Fe-2S] ISCA2, hereafter). The vacant coordination site of the latter iron ion is occupied in the complex by Cys 259 of IBA57 (corresponding to Cys 230 in the produced protein construct lacking the mitochondrial targeting sequence of 30 amino acids), since the latter Cys is required to form the cluster-mediated [2Fe-2S] ISCA2-IBA57 hetero-complex (as it occurs with Cys 79, Cys 144, Cys 146 of ISCA2)^21^. The IBA57 and [2Fe-2S] ISCA2 structures were employed as input to obtain the [2Fe-2S] ISCA2-IBA57 complex using a data-driven docking HADDOCK approach^36,37^. The latter approach requires ambiguous interaction restraints (AIRs) to successfully drive the protein-protein docking. To obtain AIRs, we used a bioinformatics approach that was already used by us in predicting protein-protein interfaces of metal-driven protein-protein complexes^34^. Briefly, the interface prediction program WHISCY^38^ was used to obtain the residues predicted to be involved in the protein-protein interface of the [2Fe-2S] ISCA2-IBA57 complex (Supplementary Table S1 and Fig. 3) and directly extracting the consequent AIRs provided by the program as an output. In the HADDOCK calculations, unambiguous interaction restraints between the iron ions and the iron coordinating cysteines of both proteins were also used (for details see Methods). The calculated models have been clustered on the basis of common contacts following standard HADDOCK scoring approach^39^ (Supplementary Table S2, for details see Methods). The obtained clusters of structural models can be grouped in two families (*a* and *b*) on the basis of the RMSD among the various clusters using a threshold of ~2 Å (Fig. 4). The backbone RMSD values of *a* and *b* families are 1.01 and 1.28 Å, respectively, (Supplementary Fig. S1); the two cluster families differ of ~10 Å (Fig. 4), as a consequence of a ~180° rotation around z-axis of one protein to the other in the complex, as it can be observed by comparing the best model of each family (Supplementary Fig. S2). In both families, the [2Fe-2S] cluster is buried at the interface of the two proteins (Fig. 5). In the overall best scoring model of family *a*, protein-protein recognition is stabilized by two intermolecular electrostatic interactions (Asp 111 (ISCA2) with Arg 146 (IBA57) and Glu 126 (ISCA2) with Arg 268 (IBA57)) which surround the cluster on opposite sides and by few hydrophobic interactions involving four residues at the protein-protein interface (Fig. 5a). The interaction involves a relatively small surface of ~1400 Å^2^, a value typically found in metal-mediated protein-protein interactions^40,41^. In family *b*, electrostatic and hydrophobic contacts as well as the buried surface area value are similar to those of family *a* (Fig. 5b), thus explaining why both structural arrangements are similarly favoured (Supplementary Table S2). Indeed, comparing the two families, the same two arginine residues of IBA57 (Arg 146 and Arg 268) are involved in electrostatic interactions with Glu 126 (in family *b*) or Asp 111 (in family *a*) and with Glu 75 (in family *b*) or Glu 126 (in family *a*) of ISCA2, respectively (Fig. 5 and Supplementary Fig. S1), and a similar hydrophobic patch involving the same ISCA2 residues in both structural models, i.e. Leu 127 and Ile 128 are present at the protein-protein interface of both families (Fig. 5). In particular, the electrostatic interaction involving Arg 146 of IBA57 shows that the average distance between the two oppositely charged residues is significantly shorter in family *b* than in family *a*, providing a potential criterion for discriminating between the two families of structures. In conclusion, two structural models for the [2Fe-2S] ISCA2-IBA57 complex with comparable HADDOCK scores were obtained through the bioinformatic prediction-driven modelling approach.

SAXS data collected on the [2Fe-2S] ISCA2-IBA57 complex (Supplementary Fig. S3) showed essentially no concentration effect. The overall parameters calculated from the SAXS curve (Table 1) clearly pointed to an oligomerization state larger than a heterodimer in these experimental conditions, and indeed a rigid-body modelling assuming a heterodimer did not satisfactorily fit the data. The two best scoring dimer models from family *a* and *b* were thus used for the rigid-body modelling against the SAXS curve of the [2Fe-2S] ISCA2-IBA57 complex assuming a dimer of dimers (see *Methods*). The resulting prolate dimers of dimers are similar in the overall shape for both dimeric models from family *a* and *b*, and fit the data with similar quality (final χ2 1.314–2.118 for family *a* 1 and 1.332–2.268 for family *b*). A subset of the solutions features an ISCA2 homodimer core (Fig. 1c χ2 = 1.407). Interestingly, the core ISCA2 dimer extracted from this model also fits reasonably well the SAXS profile of the isolated ISCA2 homodimer, as displayed in Supplementary Fig. S4. The arrangement of ISCA2 at the core of the dimer of dimers with respect to IBA57 is somewhat less compact for the rigid body models obtained employing the best scoring heterodimer from family *b* than that from family *a* (Supplementary Fig. S5). Another subset of rigid body models, both for family *a* and *b*, feature the ISCA2 molecules at the periphery, with IBA57 providing the homodimerization interface (Supplementary Fig. S6). Also these models are also in agreement with the SAXS data collected on the complex. Moreover, the orientation and the dimerization interface of ISCA2 may of course differ in the apo ISCA2 homodimer and in the dimer of [2Fe-2S] ISCA2-IBA57 heterodimers. On the other hand, performing *ab initio* multiphase modelling (see *Methods*) for a prolate dimer of dimers fitting simultaneously the SAXS curves from the ISCA2 homodimer and from the complex, it results that the obtained model features ISCA2 at the core of the assembly (Fig. 1d, χ2 = 1.197 for the curve of the complex and 0.961 for the curve of the ISCA2 homodimer). Thus, *ab initio* modelling indicates that the formation of the dimer of dimers assembly *via* ISCA2 can be considered more probable than the dimer of dimers assembly *via* IBA57.

### Molecular grounds of the pathogenic impact of the aminoacid exchange Arg146Trp in IBA57

**Figure 6:**
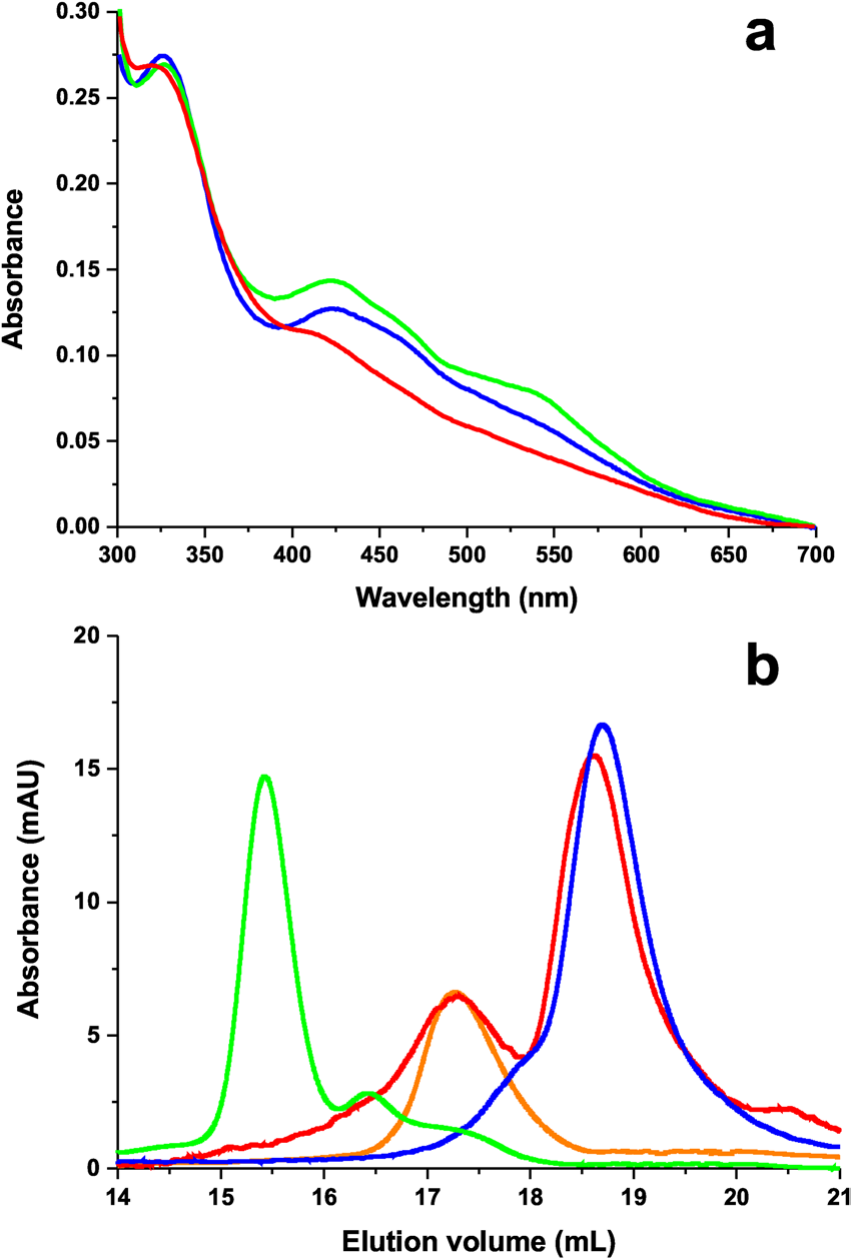
The pathogenic Arg146Trp mutation in IBA57 abolishes complex formation with ISCA2. (**a**) UV-visible spectra of the Fe-S chemically reconstituted 1:1 Arg146Trp IBA57/apo ISCA2 mixture (red), [2Fe-2S] ISCA2 (blue) and the [2Fe-2S] ISCA2-IBA57 hetero-complex (green). (**b**) Elution profiles obtained for Arg146Trp IBA57 (blue), dimeric apo ISCA2 (orange), the [2Fe-2S] ISCA2-IBA57 hetero-complex (green) and a Fe-S chemically reconstituted 1:1 mixture of Arg146Trp IBA57 and apo ISCA2 (red), obtained by gel filtration on analytical Superdex 200 Increase 10/300 GL column. mAU: milli absorbance unit.

Some of the WHISCY-identified residues of IBA57 (Arg105Trp, Arg146Trp, Val253Leu, Ile261Thr) and ISCA2 (Gly77Ser) located at or around the protein-protein interface in the complex, are mutated in patients affected by multiple mitochondrial dysfunction syndromes^22–28,42,43^. Among them, Arg 146 is the most solvent-exposed and is located close to Cys 259 ligand, suggesting that it can play a role in the [2Fe-2S] ISCA2-IBA57 complex formation. The pathogenic mutation of Arg 146 to Trp does not significantly affect the stability of IBA57 *in vivo*^22^, at variance with what previously described in other pathogenic missense mutations^26–28^, but however impairs its function. All together, these data strongly suggest that the Arg 146 mutation affects IBA57 function because of its altered interaction with ISCA2. We have therefore investigated such hypothesis, by producing the Arg146Trp IBA57 mutant and testing its interaction with ISCA2. We found that the two proteins do not interact with each other in their apo form similar to what found for the wild-type proteins. Upon chemical reconstitution of a 1:1 Arg146Trp IBA57/apo ISCA2 mixture with Fe-S cluster, no hetero-complex was formed. Indeed, the UV-vis spectrum recorded on the latter mixture compares well with that of [2Fe-2S] ISCA2 and not with that of the chemically reconstituted [2Fe-2S] ISCA2-IBA57 complex. Specifically, the band at 540 nm, which is a marker of the formation of the [2Fe-2S] ISCA2-IBA57 hetero-complex^21^, is not present in the UV-vis spectrum of the chemically reconstituted ISCA2-Arg146Trp IBA57 mixture (Fig. 6a). Consistent with no complex formation, the analytical gel filtration of the latter mixture did not show the presence of any peak at elution volumes higher than those of monomeric Arg146Trp IBA57 and dimeric apo ISCA2 as well as a peak at the elution volume of the wild-type hetero complex is not present (Fig. 6b). This result is in agreement with the HADDOCK structural models (families *a* and *b*) which showed, indeed, that Arg 146 is involved in an electrostatic interaction at the protein-protein interface (Fig. 5). In particular, the observation that this electrostatic interaction is stronger in family *b* than in family *a* (see above) and that its disruption by Arg146 mutation abolishes complex formation suggests that family *b* is the favorite structural organization of the [2Fe-2S] ISCA2-IBA57 hetero-complex. In conclusion, the pathogenic effects of the Arg146Trp mutation^22^ are likely a consequence of the impairment of the complex formation between IBA57 and ISCA2. This conclusion supports the proposal that the [2Fe-2S] ISCA2-IBA57 hetero-complex is a physiologically relevant species playing a role in mitochondrial [4Fe-4S] protein biogenesis.

## Discussion

Several *in vivo* and *in vitro* data strictly associate human IBA57 and ISCA2 as proteins working in late steps of the ISC assembly machinery. Patients with pathogenic IBA57 mutations, that markedly reduce the protein levels of IBA57 in fibroblasts mitochondria, displayed expression levels of ISCA2 drastically decreased in all the patients^26^, as well as ISCA2 patients have a decreased amount of IBA57^32^. Proteomic studies indicated that ISCA2 specifically interacts with IBA57 and not with ISCA1^20^. This specific ISCA2-IBA57 interaction is helpful to rationalize the effects that the pathogenic mutations in IBA57 and ISCA2 have, i.e. the reduced protein levels of ISCA2 and IBA57 respectively induced by IBA57 and ISCA2 mutations can be interpreted as a lack of hetero-complex formation. Finally, we have recently described an *in vitro* [2Fe-2S] cluster transfer pathway involving ISCA2, IBA57 and GLRX5, which leads to the formation of a [2Fe-2S] ISCA2-IBA57 complex, which is resistant to highly oxidative environments and is capable of reactivating apo aconitase^21^.

To gain structural insights on the stringent functional association characterizing IBA57 and ISCA2 proteins, an integrative approach utilizing information from SAXS and bioinformatics-driven docking prediction was used to determine a low-resolution structural model of the human mitochondrial [2Fe-2S] ISCA2-IBA57 complex. SAXS data on IBA57 and apo ISCA2 showed that the two proteins are, respectively, monomeric and dimeric in solution, in agreement with previous studies^18,21^. The SAXS data are in agreement with the solved crystal structure of IBA57^21,33^, indicating that this structural arrangement is preserved in solution. Since no structure is available for dimeric apo ISCA2, structural models were obtained either by SAXS-guided rigid-body modeling of the dimer of ISCA2 from monomeric ISCA2 structural model or by imposing the same dimerization as in the crystal structures of bacterial ISCA homologues. These models were used to interpret the SAXS data of apo ISCA2. All the fits led to two modes of ISCA2 dimerization, both sharing a “8”-like shape but with different dimeric interfaces (solvent exposed *vs*. facing cysteines, Fig. 2a,b), and with fits of similar quality to the experimental SAXS profiles, thus preventing a conclusive definition of the precise orientation of the ISCA2 subunits in the dimer. On the other hand, apo ISCA proteins are known to assume different dimerization modes as observed in bacterial ISCA protein structures, such as that of the crystal structures of *E. coli* IscA^44,45^. Thus, assuming that the two dimerization modes (solvent exposed *vs*. facing cysteines, Fig. 2a,b) are both possible in human apo ISCA2 as it occurs for bacterial apo ISCAs, we may suggest that the “facing cysteines”, SufA-like, dimer is formed to allow the binding of the Fe-S cluster at the subunit-subunit interface of the ISCA2 homodimer. This is, indeed, supported by the fact that it has been previously proposed, on the basis of NMR data^18^, that apo ISCA2 dimerizes through the same inter-subunit contact region observed in SufA. The apo ISCA2 dimer, which exposes the cysteine of the two subunits on the opposite sides is, on the contrary, a structural arrangement suitable for sharing the Fe-S cluster, coordinated by three ISCA2 cysteines, with a partner protein which then donates the fourth cysteine ligand, as it occurs in the ISCA2-IBA57 complex. We have recently proposed that reductive coupling of two, GLRX5-donated, [2Fe-2S]^2+^ clusters to form a single [4Fe-4S]^2+^ cluster on homodimeric ISCA2, requires six Cys ligands (three for each ISCA molecule) facing each other to promote the [4Fe-4S] cluster formation^19^. On this basis, we can assume that the facing cysteines structural model of ISCA2 dimer (Fig. 2b) and possibly also of the ISCA1-ISCA2 dimer^18,46^ is able to assemble a [4Fe-4S] cluster, while the ISCA2-IBA57 complex with only four cysteines available cannot assemble a [4Fe-4S] cluster on its own by receiving two [2Fe-2S] clusters form GLRX5, but can only receive from GLRX5 and binds a [2Fe-2S] cluster^21^. On the other hand, considering i) the genomic data correlating IBA57 protein family to oxidative stress^47,48^, ii) the stability of the [2Fe-2S] cluster against oxidative degradation, when bound to the ISCA2-IBA57 complex with respect to that bound to ISCA2 alone^21^, and iii) the ability of the [2Fe-2S] ISCA2-IBA57 complex to reactivate *in vitro* the oxidatively damaged [4Fe-4S] aconitase^21^, we can suggest that the [2Fe-2S] ISCA2-IBA57 heterocomplex can play a role in forming/repairing [4Fe-4S] clusters under aerobic cellular conditions by transferring one/two [2Fe-2S] clusters to mitochondrial target proteins which directly generate the [4Fe-4S] cluster. In conclusion, it might be possible that two alternative [4Fe-4S] assembly pathways, i.e. ISCA1-ISCA2- and IBA57-ISCA2-related pathways, are operative upon specific cellular conditions. The possible presence of the two alternative pathways is in agreement with the strict functional association found between ISCA1 and ISCA2 and between ISCA2 and IBA57, but not between ISCA1 and IBA57. Indeed, both *in vivo* and *in vitro* data showed that ISCA1 and ISCA2 interact each other and only ISCA2, and not ISCA1, interacts with IBA57^18,21,46^. Moreover, ISCA2 and IBA57 pathogenic mutations markedly reduce the protein levels of IBA57 and ISCA2, respectively, in the patients^26,32^, while the protein levels of ISCA1 are not affected or even slightly overexpressed in the patients affected by IBA57 pathogenic mutations^26^. The latter effect is in agreement with studies in HeLa cells where silencing of IBA57 was associated to unchanged or increased expression levels of ISCA1^14^.

**Figure 7:**
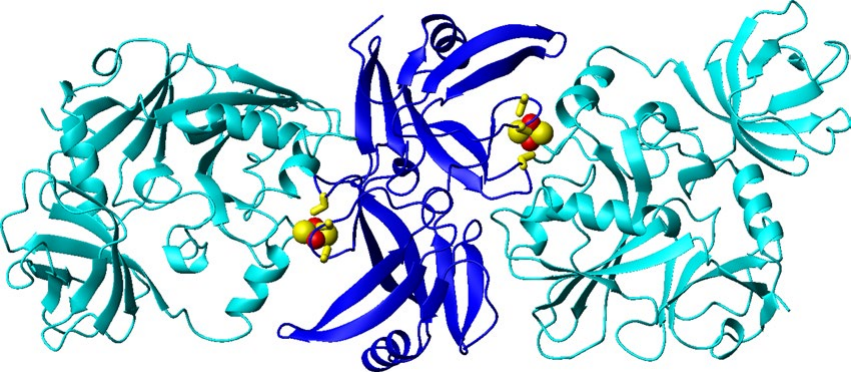
Structure of the dimer of dimers of [2Fe-2S] ISCA2-IBA57. The structure was obtained by rigid-body modelling of the [2Fe-2S] ISCA2-IBA57 complex against the SAXS curve of the [2Fe-2S] ISCA2-IBA57 complex using the best scoring dimeric model of family *a*. IBA57 and ISCA2 proteins are in cyano and blue, respectively. [2Fe-2S] clusters and Cys ligands are shown in CPK and stick modes, respectively.

The SAXS data on the [2Fe-2S] ISCA2-IBA57 heterocomplex point to an oligomerization state larger than a heterodimer, being nicely modelled *ab initio* by a dimer of dimers with prolate anisometry and featuring density for the ISCA2 homodimer at its core. The WHISCY-HADDOCK derived structural models of the heterodimeric [2Fe-2S] ISCA2-IBA57 complex (family *a* and family *b*) well fit with the SAXS data, with a subset of solutions featuring the ISCA2 homodimer at its core, in agreement with the *ab initio* modelling. Furthermore, the ISCA2 homodimer core taken from the rigid-body modelling of the complex (obtained by family *a* model) also fits the SAXS profile of the ISCA2 homodimer. We can thus reasonably propose that the dimeric ISCA2 in the complex exposes the three cysteine ligands on opposite sides in order to share the Fe-S cluster with IBA57, i.e. in a structural arrangement reproducing the “solvent exposed cysteines” dimer of apo ISCA2. The structure of the dimer of dimers of the [2Fe-2S] ISCA2-IBA57 complex, obtained by rigid-body modelling the SAXS data of the complex with family *a* model, shows that the [2Fe-2S] cluster is, indeed, out of the ISCA2 core and shared with IBA57 (Fig. 7). We regard this assembly as the most consistent with all the available experimental data.

The dimer of dimers structural organization found in the [2Fe-2S] ISCA2-IBA57 differs from what we previously observed by running SEC-MALS on the [2Fe-2S] ISCA2-IBA57 complex^21^. The latter elutes in SEC-MALS with a molar mass of 51.2 ± 0.5 kDa, corresponding to a heterodimeric complex formed by one monomeric ISCA2 (12565 Da) and one monomeric IBA57 (34925 Da). This different behaviour can be rationalized considering the different experimental conditions used to obtain SEC-MALS *vs*. SAXS data, i.e. the on-line size-exclusion chromatography was indeed avoided in order to reach the protein concentration required to run high quality SAXS data of the complex. Thus, it appears that both protein concentration and size-exclusion chromatography conditions affect the oligomerization state of the [2Fe-2S] ISCA2-IBA57 complex. Consistently, more and more NMR signals are observed in ^1^H-^15^N HSQC spectra acquired on [2Fe-2S] ^15^N-labelled ISCA2-unlabelled IBA57 upon decreasing complex concentrations (Supplementary Fig. S7). This suggests the presence of an equilibrium in solution between higher and lower molecular weight forms. The dimer of dimers is favoured at high concentrations of the complex and its signals are largely undetectable in the ^1^H-^15^N HSQC spectrum, while the heterodimeric complex is favoured at low complex concentrations, whose signals became detectable in the ^1^H-^15^N HSQC spectrum. Therefore, all these data indicated that the [2Fe-2S] ISCA2-IBA57 complex is preferentially present in diluted solutions as heterodimer and that the latter has a high tendency to dimerize via the interaction of two ISCA2 molecules of two heterodimers. These results support the proposal that the dimer of dimers is induced by the *in vitro* conditions used in SAXS measurements and that the heterodimeric species is most likely the physiologically relevant species. In support to this model, we found that the WHISCY approach applied to define the ISCA2-IBA57 interaction identified on ISCA2 only the interaction surface involving the conserved cysteine cluster binding ligands, i.e. that surface interacting with IBA57. No other interaction regions were predicted by WHISCY. Therefore, WHISCY approach does not provide any clues on the residues at the interface of the two ISCA2 molecules in the dimer of dimers observed by SAXS. Thus, this result supports that the ISCA2-ISCA2 interaction in the dimer of dimers is not a physiologically relevant species. On the other hand, the dimer of dimers formation might reflect a physiologically relevant tendency of ISCA2 to potentially dimerize with its physiological ISCA1 partner to form a ternary complex composed by [2Fe-2S] ISCA2-IBA57 and ISCA1. No data are, however, still available showing the formation of this ternary complex.

Our study provided a major contribution to understand the pathogenic role of a mutation on IBA57, Arg146Trp, among the twenty-five IBA57 mutations that have been up to now reported in multiple mitochondrial dysfunction syndrome 3^24^. Among them, seventeen are missense mutations and eight are truncating mutations^24^. Displaying all the missense residues on the IBA57 structure (Supplementary Fig. S8), it results that the majority of them (twelve out of seventeen) are part of the protein core with very low solvent accessibility (with relative solvent accessibility lower than 25%) and thus not involved in protein-protein interactions. *In vivo* data, when available, showed that these pathogenic mutants have low IBA57 protein levels^26–28^. We can interpret this effect considering that the mutations of these residues largely affect protein stability as they are involved in intramolecular interactions, responsible of the correct fold of IBA57.Consequetnly, these mutations produces protein degradation in the cell. This effect was demonstrated for the first discovered pathogenic mutation of IBA57 gene^27^, i.e. Gln314 Pro, whose relative solvent accessibility is 23%. On the contrary, it was found that the pathogenic mutation of Arg 146 to Trp does not significantly affect the stability of IBA57 *in vivo*^22^. The conserved residue Arg 146 is highly solvent accessible (Fig. 7) and its mutation clearly does not destabilize the protein overall structure. The Arg146Trp mutant displays, indeed, a 1D ^1^H NMR spectrum typical of a folded protein and similar to that of the wild-type protein (Supplementary Fig. S7). The pathogenic impact of Arg mutation, being on the surface of the protein, originates from an impaired partner recognition. Indeed, Arg 146 is involved in an intermolecular electrostatic interaction with Asp/Glu of ISCA2 in our structural models and its mutation to Trp prevents complex formation. In conclusion, our structural data define the molecular grounds of the pathogenic impact of the aminoacid exchange Arg146Trp in IBA57, i.e. the function of IBA57 in mitochondrial [4Fe-4S] protein biogenesis is impaired by this mutation because of the abrogation of the [2Fe-2S] ISCA2-IBA57 complex formation. This result strongly claims on the functional relevance of the complex.

## Methods

### Protein production

IBA57 and ISCA2 proteins were obtained as previously reported^18,21^. The [2Fe-2S] ISCA2-IBA57 was obtained by chemically reconstituting with FeCl_3_ and Na_2_S salts an apo ISCA2 and IBA57 mixture as previously reported^21^.

### Mutagenesis and production of Arg146Trp pathogenic mutant

Arg146Trp mutant of human IBA57 was obtained through site-directed mutagenesis, using the pETDuet-1 vector containing the *IBA57* gene as a template. The Arg146Trp mutant of human IBA57 was expressed and purified following the same protocol applied to the wild-type protein.

### Analytical gel filtration and UV-Visible

Purified samples were loaded on a Superdex 75 HR 10/30 analytical column. Degassed 50 mM phosphate buffer (pH 7.0), 150 mM NaCl and 5 mM DTT was used as an eluent at a flow rate of 0.65 mL/min. UV-visible spectra were performed in degassed 50 mM phosphate buffer at pH 7, 5 mM DTT and 150 mM NaCl on a Cary 50 Eclipse spectrophotometer.

### SAXS data acquisition and analysis

Synchrotron SAXS data were collected at the EMBL beamline P12^49^ at the PETRA III storage ring of DESY (Hamburg, Germany). The data on IBA57 and apo ISCA2 and were collected in on-line SEC-SAXS mode, while the data on the [2Fe-2S] ISCA2-IBA57 complex were collected using an automatic sample changer, flowing the sample during the exposure to avoid radiation damage. All samples were exposed in a flow capillary (quartz, 1.7 mm or 0.9 mm). The parameters of both data collections are shown in Table 1. For SEC-SAXS, a buffer consisting of 50 mM phosphate pH 7.0, 150 mM NaCl, 5 mM DTT and 3% (v/v) glycerol was used to separate the samples over a Superdex 200 Increase 10/300 column at a flow rate of 0.5 mL/min (IBA57) or 0.75 mL/min (apo ISCA2). The SAXS curves of on IBA57 and apo ISCA2 were obtained from the main chromatographic peak. The [2Fe-2S] ISCA2-IBA57 complex, assembled with excess IBA57 (subsequently removed by analytical gel filtration) to favour complex formation, was measured in 50 mM phosphate pH 7.0, 150 mM NaCl, 5 mM DTT at ~1.8 and ~0.9 mg/mL. As a (small) increase at low angles was observed for the more concentrated sample, likely due to some aggregation, subsequent data processing was performed using the dilute one. The scattered intensity was calibrated to absolute units using the scattering of water at 293 K.

The primary data reduction was performed using SASFLOW^50^, initial data analysis with PRIMUS^51^, overall parameters were calculated using the ATSAS suite^52^. SEC-SAXS profiles were extracted from the chromatographic peak of interest using CHROMIXS^53^.

The SAXS curves from the atomic models were computed by CRYSOL^54^, rigid body modelling was performed using SASREF/SAREFCV^55,56^, multiphase *ab initio* modelling, using MONSA^57^. The models were rendered with UCSF Chimera^58^ and PyMOL Molecular Graphics System, Version 2.0^59^.

The SASREF modeling of apo ISCA2 was repeated 10 times in P2 symmetry; SASREF modeling of the complex employing the computationally generated [2Fe-2S] ISCA2-IBA57 heterodimers was repeated 20 times in P2 symmetry for both families *a* and *b* dimers.

The two-phase MONSA model was calculated by fitting simultaneously the SAXS curves of the ISCA2 homodimer and of the complex in the *s*-range of the data [0.1, 2.5 nm^−1^]. Here, one phase represented the IBA57 moiety, the other phase the ISCA2 moiety. The modeling was repeated 10 times in P2 symmetry.

### Structural coordinates

Structural model of human IBA57 was already available in the Protein Data Bank (PDB ID 6QE3)^21,33^. An experimentally validated structure of monomeric apo ISCA2 was already available^18^. Structural models for human dimeric apo ISCA2 were generated with Modeller 9.20^60^ (https://salilab.org/modeller/) using the templates *E. coli* apo IscA (PDB ID 1R95 and 1S98)^44,45^, *E. coli* apo SufA (PDB ID 2D2A)^61^, while the structural model of monomeric [2Fe-2S] ISCA2 was generated with Modeller 9.20 using as template the *T. elongates* [2Fe-2S] IscA structure (PDB ID 1 × 0 G)^35^. The [2Fe-2S] cluster bound to monomeric ISCA2 was included in the docking calculations by covalently linking one iron atom of the [2Fe-2S] cluster to the Sγ atom of Cys 79 of ISCA2. [2Fe-2S] cluster was introduced with a total charge of +2; for the four cysteine ligands, the SH hydrogen was removed and the partial charges for side chain atoms were set to +0.18 for CB and −0.68 for SG. In this way, the total net charge of four cysteines plus the [2Fe-2S] cluster is zero.

### Interface prediction

WHISCY program was used for interface predictions^38^. Multiple sequence alignments were constructed by first finding homologues of the human ISCA2 and IBA57 proteins using blastp against the nr database using default settings^62^, except that a restriction to only eukaryote sequences was used since the Fe-S cluster-delivery pathway might be different in prokaryotes as compared to eukaryotes. Subsequently, multiple sequence alignment was performed using ClustalW1.8^63^. Interface predictions were used to generate AIRs. Specifically, active residues are defined based on interface predictions using WHISCY, passive residues are surface neighbors of active residues defined in the HADDOCK run (Supplementary Table S1).

### Fe-S cluster-based restraints

We defined unambiguous interaction restraints between the iron atoms and the Sγ atoms of the coordinating cysteines on both ISCA2 and IBA57. The restraint distance was 2.3 Å; residues involved in those restraints are listed in Supplementary Table S1.

### Docking protocol

Structural models of the [2Fe-2S] ISCA2-IBA57 complex were calculated using the protein-protein docking program HADDOCK 2.2 (high ambiguity driven protein-protein docking) by following the standard HADDOCK procedure^36,37^. HADDOCK uses AIRs defined from any available information about the interface. In our case, active residues were based on interface prediction by WHISCY, and included as AIRs in the docking calculations as mentioned above. In addition to these AIRs, experimentally based unambiguous interaction restraints (Fe-S cluster-based restraints) deriving from previous mutagenesis data^21^ were included. The final ensemble of 200 solutions was ranked based on the HADDOCK score with standard weights^64^. The water-refined models were clustered based on the default fraction of common contacts, FCC = 0.75, with the minimum number of elements in a cluster of 4^39^. NACCESS is the program used to calculate the atomic and residue accessibilities from a PDB format file. The models were rendered with MOLMOL program^65^.

## Supplementary information

Supplementary information

## Data Availability

The authors confirm that the docking data are available from the corresponding author on request, and the SAXS models and the corresponding data are being submitted to the SASBDB, with accession codes SASDGD2 (IBA57), SASDGE2 (ISCA2), SASDGF2 (complex).
