# Supplementary material for: Structural properties of [2Fe-2S] ISCA2-IBA57: a complex of the mitochondrial iron-sulfur cluster assembly machinery
Source: Sci Rep. 2019 Dec 12;9:18986. doi: 10.1038/s41598-019-55313-5 (PMC6908724; doi:10.1038/s41598-019-55313-5)

Supplementary Information

Structural properties of [2Fe-2S] ISCA2-IBA57: a complex of the mitochondrial iron-sulfur cluster assembly machinery

Veronica Nasta[1,2,†], Stefano Da Vela[3,†], Spyridon Gourdoupis[1], Simone Ciofi-Baffoni[1,2], Dmitri I. Svergun[3], Lucia Banci[1,2,*]

[1]Magnetic Resonance Center CERM, University of Florence, Via Luigi Sacconi 6, 50019, Sesto Fiorentino, Florence, Italy.

[2]Department of Chemistry, University of Florence, Via della Lastruccia 3, 50019 Sesto Fiorentino, Florence, Italy.

[3]European Molecular Biology Laboratory, Hamburg Outstation, EMBL c/o DESY, Notkestrasse 85, 22607 Hamburg, Germany.

†These authors contributed equally to this work.

*Corresponding Author

Supplementary Table S1. List of active and passive interface residues used for docking.[a]

**ISCA2**	
Active	Gly 78, Cys 79, Leu 127, Ile 128, Cys 146
Passive	Gly 76, Gly 77, Ser 80, Gly 81, Gln 83, Phe 123, Ser 124, Gln 125, Glu 126, Arg 129, Ser 130, Ser 131, Phe 132, Gln 133, Gly 143, Cys 144, Ser 145, Gly 147, Ser 148, Ser 151
Fe-S binding	Cys 144,Cys 146[b]
**IBA57**	
Active	Gly 72, Val 102, Gln 103, Ile 145, Arg 146, Thr 256, Gly 258, Cys 259, Glu 264, Arg 268, His 271, Met 272, Gly 273
Passive	Asp 65, Ala 67, Pro 68, Leu 71, Glu 77, Leu 78, Asn 101, Arg 105, Leu 107, His 139, Ala 141, Leu 142, Tyr 143, Arg 144, Arg 147, Lys 148, Gly 238, Leu 241, Val 253, Phe 255, Lys 257, Ile 261, Gln 263, Leu 265, Ala 267, Thr 269, His 270, Val 274, Arg 276
Fe-S binding	Cys 259

[a]Ambiguous interaction restraints were defined using active and passive residues as listed in the table, based on WHISCY predictions. Active residues are residues predicted to be involved in the interaction, and passive residues are their surface neighbors. In addition, iron-sulfur-based unambiguous interaction restraints were defined as described in Methods using the Fe-S binding residues listed in the table.

[b]The iron-sulfur cluster was included in the coordinate file of ISCA2 template by creating a special residue that covalently links Cys 79 to an iron atom of the [2Fe-2S] cluster. Therefore, iron-sulfur-based unambiguous interaction restraint linking Cys 79 to the iron atom was not required in the HADDOCK calculations.

Supplementary Table S2. Cluster statistics of the HADDOCK docking run for the heterodimeric [2Fe-2S] ISCA2-IBA57 complex.[a]

CLUSTER	HADDOCK score (a.u)	Cluster population	RMSD from the overall lowest energy structure (Å)	Van der Waals energy (Kcal mol^{-1})	Electro-static energy (Kcal mol^{-1})	Desolvation energy (Kcal mol^{-1})	Restraints violation energy (Kcal mol^{-1})	Buried Surface Area (Å^2)
1	-109.8 ±13.95	48	1.085 ±0.495	-68.0 ±6.07	-301 ±54.3	-14.9 ±6.36	31.4 ±14.4	1516 ±99.23
2	-78.82 ±4.007	45	10.6 ±0.370	-35.0 ±4.61	-395 ±24.5	-9.62 ±6.72	53.4 ±18.3	1182 ±51.71
3	-91.89 ±9.292	20	11.1 ±0.161	-46.3 ±3.69	-444 ±38.4	-6.89 ±6.69	57.8 ±23.3	1175 ±90.01
4	-85.96 ±8.574	13	1.576 ±0.283	-54.3 ±4.65	-231 ±41.7	-12.3 ±3.74	37.8 ±28.5	1386 ±83.02
5	-58.25 ±11.47	10	10.2 ±0.200	-24.5 ±5.5	-343 ±40.5	-5.76 ±8.47	63.1 ±19.9	1117 ±104.2
6	-77.84 ±7.961	7	2.033 ±0.209	-49.6 ±3.51	-295 ±62.4	-0.62 ±3.64	18.5 ±31.8	1125 ±60.08
7	-79.65 ±13.42	6	1.670 ±0.291	-52.5 ±8.68	-260 ±28.0	-4.047 ±5.21	28.9 ±24.7	1287 ±135.8
8	-78.74 ±21.78	6	10.7 ±0.224	-41.8 ±10.8	-410 ±77.0	-0.64 ±8.09	47.7 ±40.6	1120 ±146.1
9	-57.32 ±6.918	5	10.6 ±0.523	-24.3 ±5.88	-314 ±72.2	-8.30 ±6.93	66.7 ±20.1	1030 ±79.94

[a]All values are averages and S.D. calculated from the best scoring ten models of each cluster.

Supplementary Figure S1. **[2Fe-2S] ISCA2-IBA57 HADDOCK models from family *a* and family *b*.** On the left, bundles of structures obtained by superimposing the backbone atoms of the 74 and 86 structures of family *a* (**a**) and *b* (**b**), respectively. ISCA2 is in red, IBA57 is in blue and the [2Fe-2S] cluster is shown in CPK mode. On the right, superimposition of the sidechain of Arg 146 and Asp 111 in family *a* (**a**) and Arg 146 and Glu 126 in family *b* (**b**). The carbon atoms of CD Asp, CG Glu and CZ Arg are shown as black spheres.

a

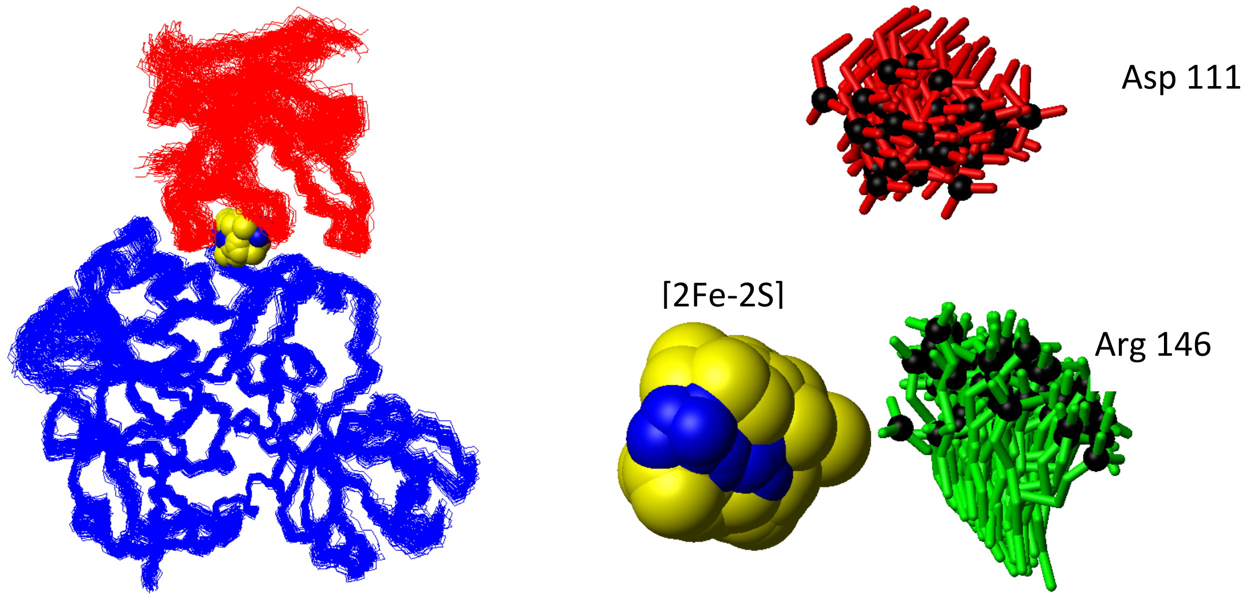

b

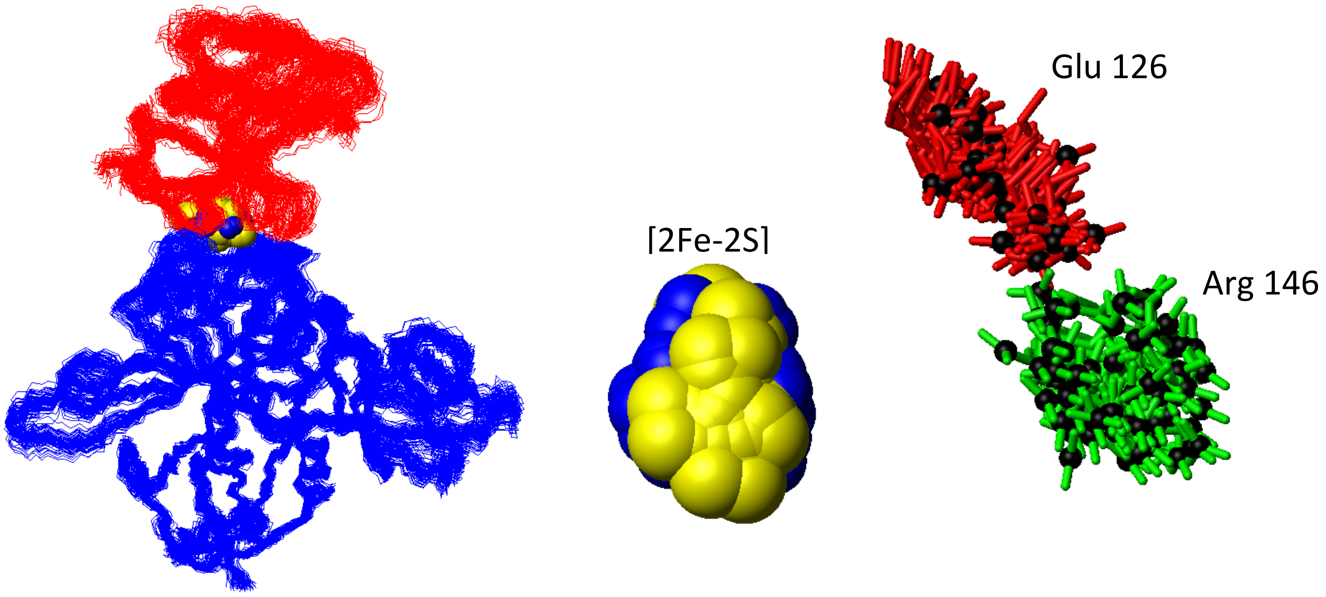

Supplementary Figure S2. **Ribbon representation of the best scoring [2Fe-2S] ISCA2-IBA57 HADDOCK models from family *a* and family *b*.** By superimposing the backbone of IBA57 proteins (in cyan) of the two models, it results that the two ISCA2 proteins (in yellow and green) are rotated of ~180° along the vertical z-axis.

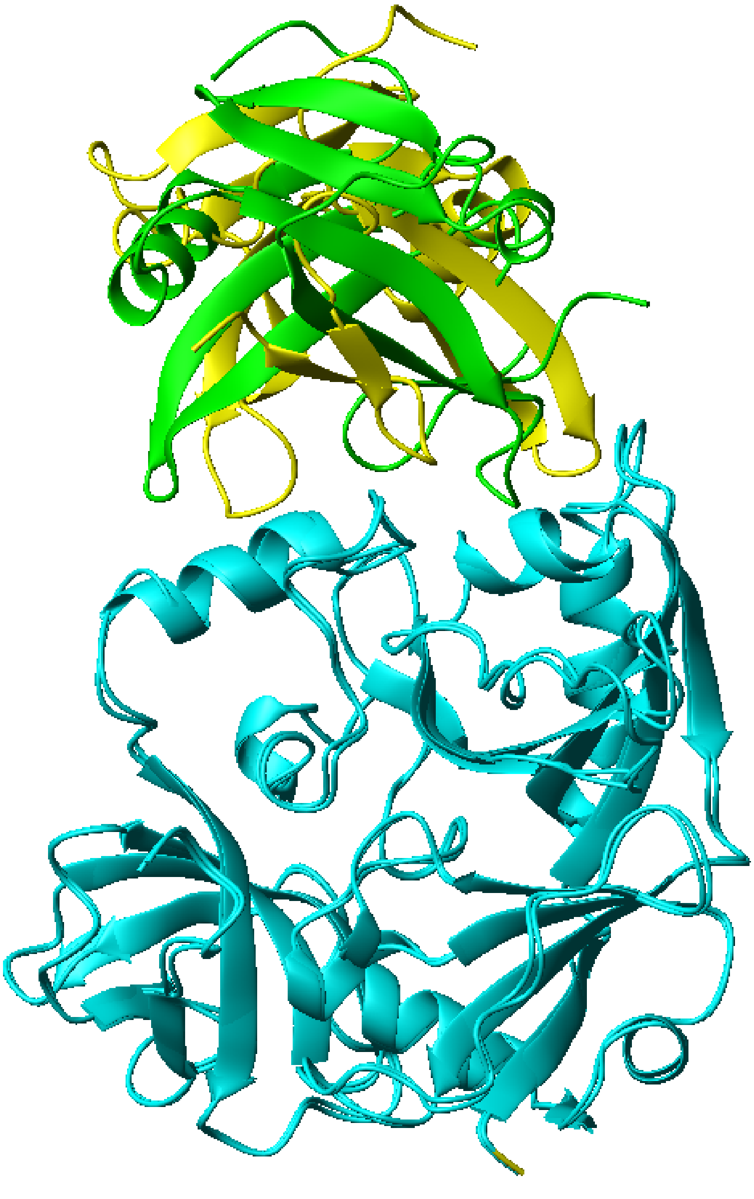

Supplementary Figure S3. SAXS profiles measured at different concentrations of the $[2Fe\text{-}2S]^{2+}$ ISCA2-IBA57 complex, in double logarithmic plot. Red: ~0.9 mg/mL. Green: ~1.8 mg/mL.

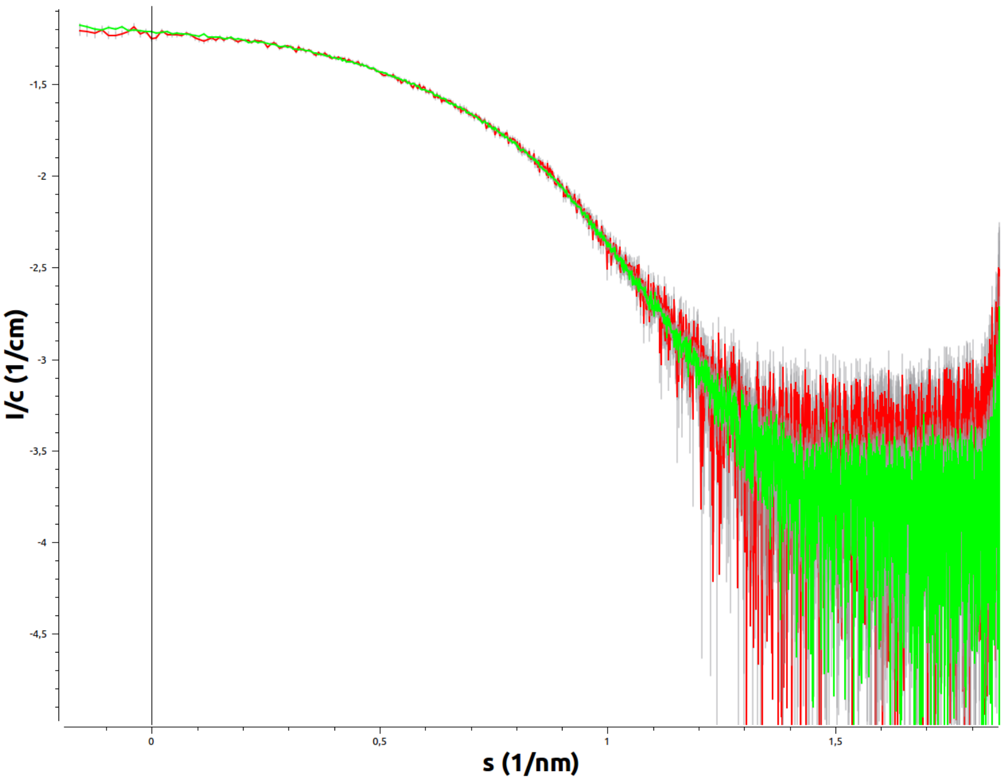

Supplementary Figure S4. Fitting the core ISCA2 dimer from the model shown in Fig. 1C, main text, to the SAXS data from the ISCA2 dimer (χ^2=1.352).

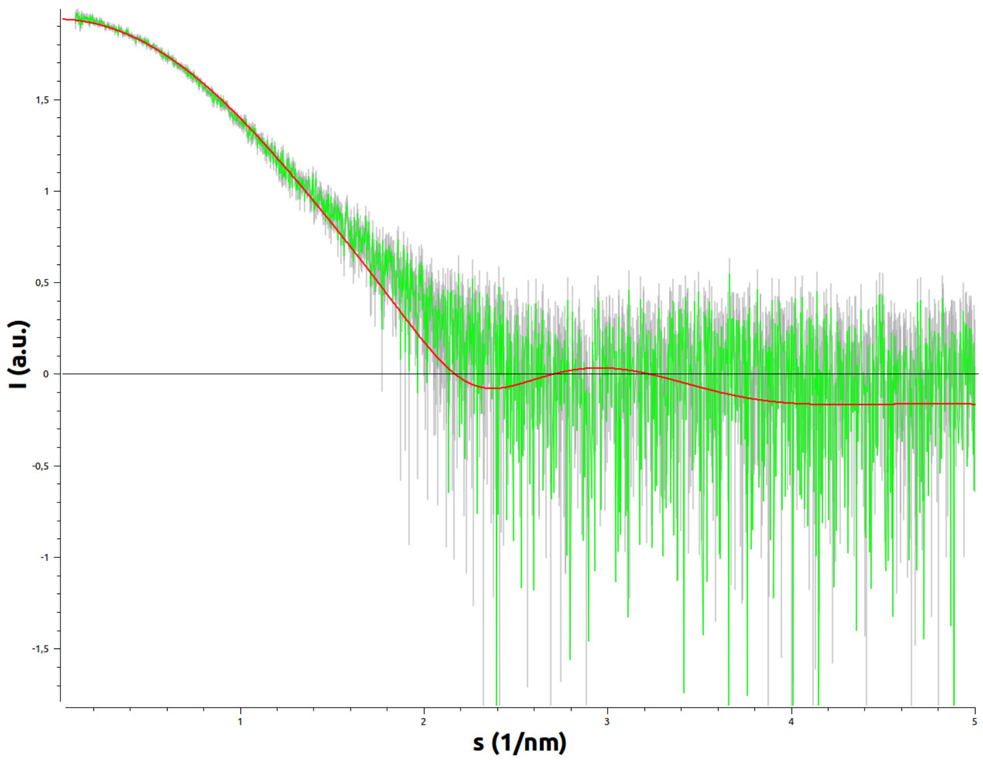

Supplementary Figure S5. Structural models of the dimer of dimers of the [2Fe-2S] ISCA2-IBA57 complex obtained by fitting the SAXS curve of the complex with the best scoring heterodimers from family *a* and *b*. (**a**) Structure from integrative rigid body modeling (χ^2=1.439) using the best scoring heterodimer from family *b*. (**b**) Structure from integrative rigid body modeling (χ^2=1.365) using the best scoring heterodimer from family *a*.

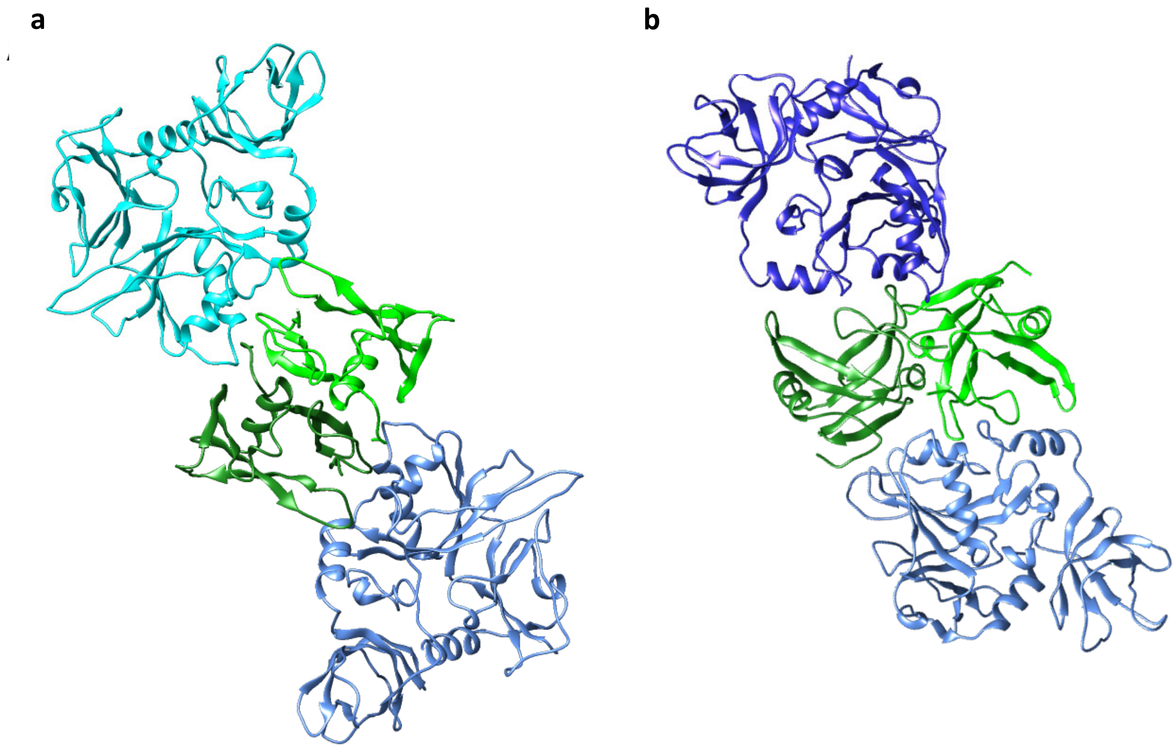

Supplementary Figure S6. Alternative model of the dimer of dimers of the [2Fe-2S] ISCA2-IBA57 complex with peripheral ISCA2 protomers. (χ^2=1.315), using the best scoring heterodimer from family *a*.

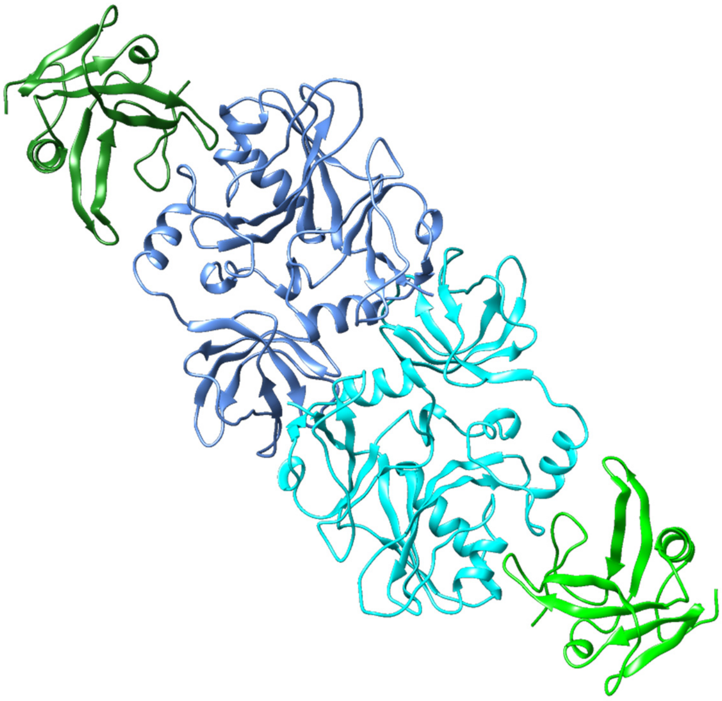

Supplementary Figure S7. **NMR data monitoring the oligomerization state of the [2Fe-2S] ISCA2-IBA57 complex and the fold of the Arg146Trp IBA57 mutant.** (**a**) Overlay of ^{1}H-^{15}N HSQC spectra of [2Fe-2S] ISCA2-IBA57 at 0.125 mM (black) and 1 mM (red) in 50 mM phosphate buffer, 5 mM DTT and 150 mM NaCl, pH 7.0 at 298 K. NMR signal intensities were normalized considering the different concentration in the two samples. The dotted black line indicates the FID row extracted from each ^{1}H-^{15}N HSQC spectrum whose FT transformed 1D spectrum is reported in panel (**b**) using the same color-code of panel **a**. (**c**) Overlay of 1D ^{1}H NMR spectra of Arg146Trp IBA57 mutant (0.15 mM) (red) and of wilde-type IBA57 (0.25 mM) (black) in 50 mM phosphate buffer, 5 mM DTT and 150 mM NaCl, pH 7.0 and 10% (v/v) D_2O at 298 K.

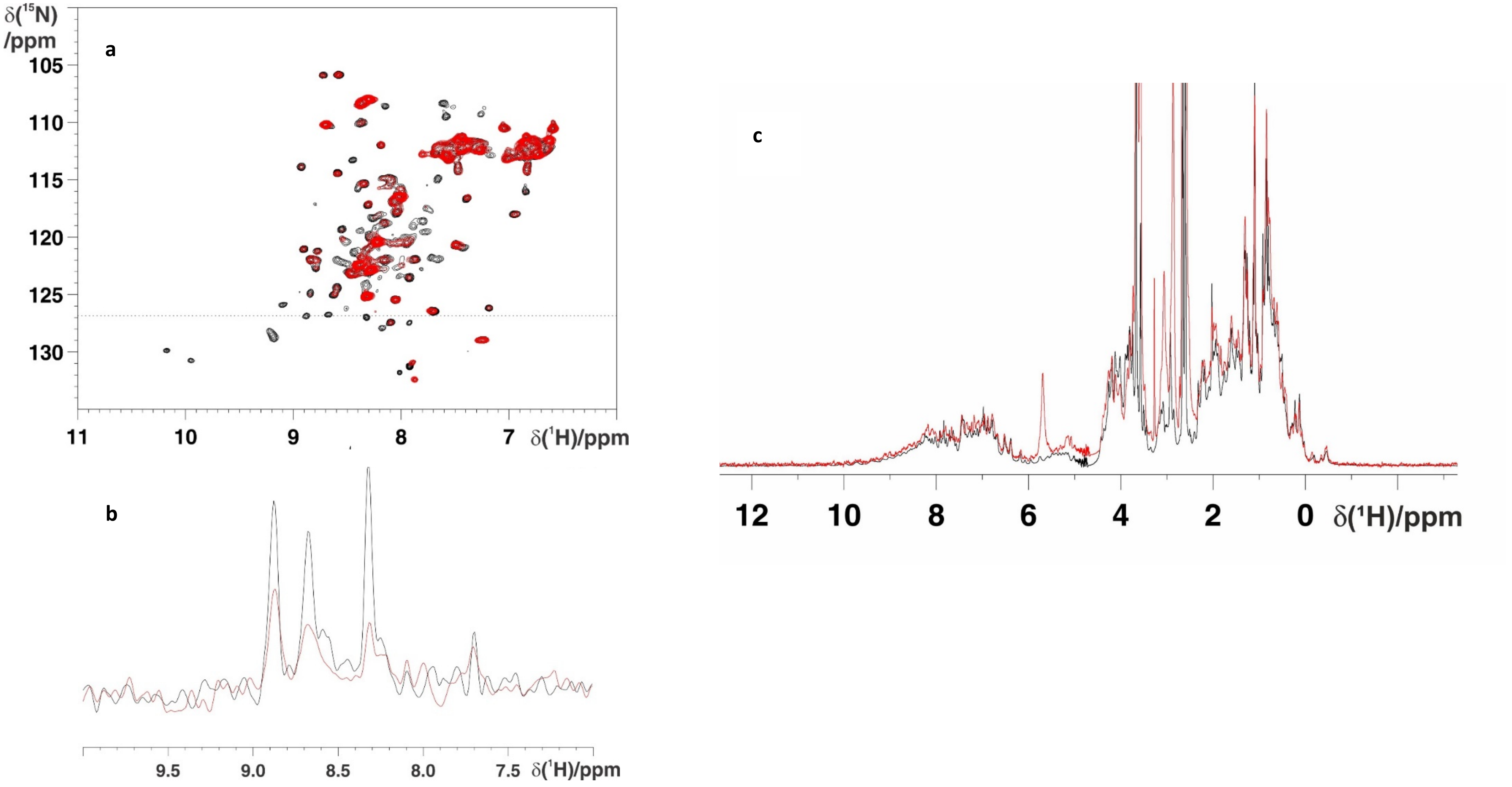

Supplementary Figure S8. **Mapping the pathogenic missense mutations on the IBA57 structure.** The seventeen residues varied in MMDS3 missense mutations are shown on the ribbon diagram of IBA57. The residues with relative solvent accessibility below 25%, calculated by NACCESS program, are in green, the other solvent accessible residues are in red or in magenta (Arg 146). The Cys 259 ligand is in yellow.

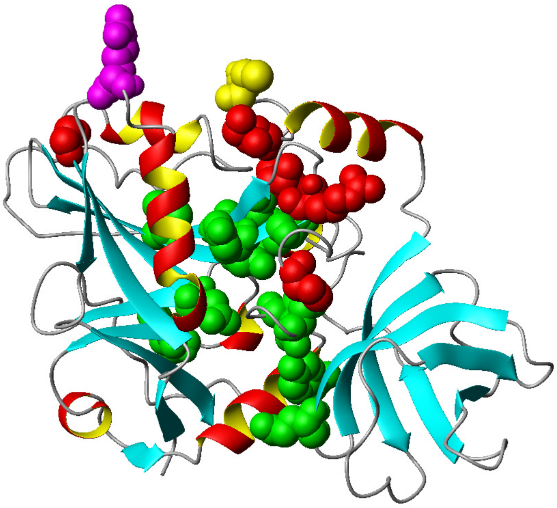

Supplement: Supplementary file 1 — Supplementary information [file 41598_2019_55313_MOESM1_ESM.pdf]
